# Foreign

**Published:** 1841-09-25

**Authors:** 


### FOREIGN.

*A Case of Luscitas Spastica, with observations.* By Aug. Franz, M. D. Leipsic, M. R. C. S., &c.—Mrs. W., aged 42, of robust and plethoric habit, has, during several years, been subject to rheumatism. In the beginning of January last she suffered from severe cold in the head, with toothache, and acute lancinating pain over the left half of the face, attended with some degree of fever. With a little care, and the use of some domestic medicines, the cold abated, and the febrile symptoms subsided; the face-ache, however, although to a certain extent diminished, was confined to the left orbit, and assumed a remittent character. It appeared principally towards evening, lasted during a part of the night, and became more intense during bad weather. At each of the pains a slight inversion of the left eye was observed, which became in time more decided and more constant. The eye was now no longer straight in the morning and during the absence of the paroxysms, as was the case at the commencement of the complaint, but remained permanently inverted. With this inversion the necessary concomitant of double vision now made its appearance; although slight in the beginning, it increased in the same ratio as the inversion, and was at last so annoying that the patient was obliged to blind one eye or the other, in order to follow her pursuits of reading, needlework, &c. When the right eye was closed, the left or squinting eye assumed always its proper position in the orbit; one morning, however, after the patient had been out to a late hour the night before, she found, on binding up the right eye, that she could no longer direct the left eye towards a book she wished to read, but the cornea was completely fixed in the inner canthus. The print appeared to her very distinct, and the inner eyelids, more especially the upper, felt stiff, and its movements were limited. The patient being much alarmed by this circumstance, called upon me a few days afterwards for advice.

On the 8th of March, when I for the first time saw her, nothing abnormal could be distinguished in the right eyeball, neither in its position nor movements; the iris even responded properly to different degrees of light. The left eye, on the contrary, was permanently fixed in an inverted position, so that one quarter of the cornea, which was not only inverted inwards, but also a little upwards, was hidden by the inner canthus and the upper lid. When the right eye was closed the left could not be brought into a proper position, but remained immoveably inverted. The pupil, although somewhat shaded by the inner canthus, was diminished in size, and did not answer to different degrees of light, nor contract even when a strong light was concentrated into it by means of a lens. On opening the eyes, the aperture between the lids was of the same extent in both eyes; but the upper lid of the left eye could only with difficulty be moved downwards, and then did not entirely cover the globe: it could, however, be brought in contact with the lower lid when drawn down with the fingers. In the inner canthus the conjunctiva was a little reddened, and the sclerotica presented a fine vascularity; the eyeball felt compressed, and as if pushed forwards, and in the orbit was perceptible a sensation of fulness and tension, with an occasional pricking and lancinating pain. There was no intolerance of light. On closing the right eye, remote objects could not be distingushed, and those near her were seen very indistinctly and faintly. When both eyes were open, the double vision was well marked.

From the history of the case, and the appearance of the affected eye, I was induced to believe that the complaint was a spasmodic affection of some of the muscles of the eye and upper lid, dependent on a rheumatic or hysteric cause, and consequently ordered the following treatment to be pursued:—Eight leeches to the temple; linseed poultice, with Herb. Hyoscyami, to the eye; a warm bath on two succeeding days; after which perspiration was promoted by means of Pulv. Ipecac. comp.; the bowels were kept gently open. On the third day the affected eye could already be moved from the inner canthus towards the centre of the orbit, when the other was closed, but returned to its inverted position on the cessation of the effort of the will. Emplast. Cantharidis was then applied behind the left ear; the eye was fomented with Infus. Anthemides et Hyoscyami; Ung. Hydr. with a little Extr. Belladonnæ rubbed into the lids; irritating foot-baths at bedtime, and Tinct. Colchici, which acted freely on the bowels, were ordered, and the left eye principally to be used. By these means the inversion and other symptoms were gradually lessened, and disappeared entirely a fortnight after the commencement of this treatment; so that at this time the eyeball had its proper position in the orbit, enjoyed freedom of motion in all directions, could be completely covered by the upper lid, and the pupil was of its proper dimensions, expanding and contracting regularly: in fact, no difference existed between both the eyes. As the sclerotica was yet somewhat vascular, and a slight sensation of tension was felt in the orbit on moving the eye, the use of Tinct. Colch. and the ointment was continued for a few days, and the patient advised never to wash the face with cold water. Under this treatment the affection soon vanished, and the eye has ever since been perfect in its position, movements, and function of vision, as the iris also in its actions.

The accomplishment of a cure under this mode of treatment in so short a space of time, proves that the permanent inversion or *luscitas* in this case was not the effect of a disease of the brain, nor of paralysis, or any other affection of the musculus rectus externus, nor of any adventitious growth in the orbit, but was of a spasmodic nature, at first remitting but subsequently permanent, of which rheumatism wasthe proximate cause. The spasmodic affection extended to the rectus internus, probably to the rectus superior and inferior, and also to the obliquus inferior, and levator palpabræ superioris, as is evident from the direction of the cornea inwards and a little upwards, and from the limited motion of the upper lid downwards. The affected muscles being only those supplied by the third pair of nerves or motores oculorum, it can hardly be doubted that the rheumatism, which at first affected the left half of the face, was in time restricted to the sheaths of the left oculo motorius exclusively. It seems moreover probable, that not only the muscular branches of this nerve were affected by rheumatism, but also the short thick branch given off to the ciliary ganglion, and that thus was occasioned the permanent spasmodic contraction of the pupillary margin of the iris, and consequently the diminished size of the pupil.

In eyes affected with inversion I have sometimes noticed the pupil to be contracted, but at other times, and indeed far more frequently, dilated. The interesting question now presents itself—how far this difference in the state of the pupil may be considered as a diagnostic sign, as regards the primary cause of the inversion? From the above case, and from some other observations I have made in this respect, it appears to me that a contracted pupil in an inverted eye would indicate that the original cause of the inversion was a spasmodic state of one or more of the muscles; for if this state continues some time, it will make the temporary contraction of the pupil and muscles of the permanent eye. Professor Müller,* on the other hand, says, "There is a certain degree of paralysis of the muscles with contraction, or also a contraction with atrophy." In club-foot, the gastrocnemius sometimes presents either the one or other of these conditions. Now, if the inversion of an eye has been caused by such conditions of the muscle, I think we may fairly expect the state of the pupil to be that of dilatation. The pupil may, however, be dilated by being much shaded by the inner canthus, when the eye is inverted to a great extent, without any reference to the existing muscular defect.—*Lond. Med. Gaz.*

---

*Glanders in the Human Subject.*—DR. HUTTON said that as four or five cases of glanders in the human subject had, within a comparatively short period, come under his own notice, or that of the surgeons of the House of Industry, he was anxious to lay them briefly before the Society, and also to exhibit a specimen of the disease as it had manifested itself in the lungs of a patient who died about two days before. Previous, however, to entering on this case he would read the details of another, in which some experiments were made with the view of testing the character of the poison, and ascertaining whether it was glanders or not. One of the results of these was, that an ass, inoculated with matter taken from the patient, was in due course attacked with the disease. The case was recorded by Mr. Rutherford, one of the resident pupils of the Hospital, for whose accuracy Dr. Hutton could vouch. The subject, a young man named P. Kelly, aged about twenty, was admitted into the Richmond Hospital on the 26th of August, 1838. On admission, his face presented that peculiar aspect which is so characteristic of glanders; the left half was very much swollen, tense and shining, the redness fading away gradually and becoming lost in the surrounding integuments. Both eyes, but particularly the left, were closed, from inflammation and œdema of the lids. The left ear was swollen, of a dark red or livid colour, and the patient was quite deaf on that side. The glands of the left side of the jaw and face were enlarged and indurated, and he complained of a feeling of numbness in the whole of that side of the head and face. About an inch and a half in front of the ear there was a large flaccid vesicle; there were also two pustules on the face, one of which had burst and was sloughing. On various parts of the body there were numerous pustules in different states, from the first to the more advanced stages. In the first stage, the skin in the situation where the vesicle afterwards occurred was of a peculiar pale, whitish appearance; in the next stage the vesicle appeared, not, however, exactly in the centre of the pale spot, but rather to one side. In a more advanced stage it became seropurulent, then pustular, and some time afterwards the pustules began to shrink and become depressed, in the centre. The mucous membrane of the mouth was inflamed and covered with a viscid adhesive mucus; the schneiderian membrane was also inflamed, but there was no discharge of purulent matter from it. The patient had the ordinary symptoms of irritative fever; his head was confused, but he had no pain or raving; his bowels rather free; his urine high colored. He stated that he had been always healthy, and when questioned as to the nature of his occupation, said that he had been employed for the last four months in attending horses which were laboring under glanders; that he had been retained specially for that purpose, and groomed the animals once a day. He did not recollect that he had a wound or a sore on either hand; he had not drank out of any vessel used by the horses, nor had he slept in the stable. He attributed his illness to fatigue after a long journey, and said that the first symptoms he had noticed were pain in his knees, followed by headache. Four days afterwards the left side of his face and head began to swell, with increase of fever and depression of strength. On the 27th, the day after admission, his symptoms were progressing; the tumefaction of the head and face increased, and several livid vesicles made their appearance, accompanied by severe pain in both jaws. Several ve-

*Physiology, vol. ii. p, 82, German edition.

sicles now began to show themselves on the anterior part of the arms and chest; his pulse became smaller, and rose to 120; his respiration was somewhat suspicious; his breath fœtid; and he felt pain when the ends of the long bones were pressed on in the vicinity of the joints. His head was still confused, but he had no raving. Towards eight o'clock in the afternoon there was a further exacerbation of his symptoms. He made water tolerably well, but did not seem to be aware of passing it. He was ordered to take ten grains of sulphate of quinine three times a day. On the 28th the eruption was still extending; his pulse 140, and weak; his thirst excessive, and he raved frequently. At half-past 3, P.M., he was restless and tossing about in bed, with constant involuntary motions of the lower extremities, quick small pulse, and hurried respiration. Twelve new spots had now made their appearance, his fever and delirium were increased, and he was passing both urine and fæces involuntarily. There was a discharge of sanious fluid from the left ear, but none from the nostril. On the 29th a further exacerbation of symptoms took place. The left elbow joint was swollen and painful; the pustules increased in number and size, and were intermixed with gangrenous bullæ; and along the internal and anterior parts of the thighs, in the situation of the absorbents, pale, rose-coloured swellings began to appear. His breath was fœtid, and the odour from his whole body almost insupportable. He had no discharge from the nostrils, but, on examining the nose, Dr. Hutton observed a small ulcer on the left side of the septum narium. The man died during the course of the night.

On examination a great number of small circumscribed abscesses or purulent depots were found in the extremities; as many as thirty were found in the left arm. There were two or three of the same kind in the pectoralis major, and several of the same description in the recti of both thighs, all circumscribed and imbedded in the muscular tissue. In the left lung there was a small depot of pus, surrounded by a dark, livid border; another of the same kind was discovered in the right lung. On the posterior surface of the heart there were dark coloured spots, and the blood was remarkably fluid in all the vessels. There was a deposition of pus under the mucous membrane of the larynx, and also on the posterior surface of the epiglottis; the left half of the face was in a semi-gangrenous state.

Shortly before this, another case of the same kind had occurred in the female ward. An ass which had been procured for the purpose, was inoculated with purulent matter taken on the fourth day from the patient. No disease was produced. On the 27th of August, the day after Kelly's admission, the experiment was again repeated on another with matter taken from the vesicles and pustules on his body. The lymph was inserted into the left nostril of the animal, the pus into the opposite one; it was also inserted into the ear. On the following evening the ass appeared unwell, and next day had enlargement of one of the glands of the jaw on the left side, with increased heat and tenderness, accompanied by feverish symptoms. The left ala nasi swelled, and the line of absorbents from this to the glands on the side of the jaw could be distinctly traced. Next day there was a profuse watery discharge from both nostrils, particularly the left; and on the following day, the fifth after inoculation, the discharge was purulent. Soon afterwards the animal was killed with nux vomica, it having been previously ascertained, by Mr. Ferguson, V. S., that the animal was really glandered.

On examination, a cluster of pustules, having a tubercular aspect, were found in the left nostril; in the right there were circular patches of ulceration. Similar ulcers were found in the interior of the stomach, and there was a cluster of pustules in the anterior lobe of one lung. There was no morbid appearance in the larynx or trachea. Dr. Hutton exhibited several drawings to show the condition of the various parts, particularly the nostrils, stomach, and lungs. The next case, to which he would merely allude, as it was about to be published by Dr. M'Donnell, who had charge of it, had occurred a short time ago at the Richmond Hospital. The patient was admitted for an ac cident of which he recovered, but before he left the hospital he was seized with an affection of the joints, followed by an eruption of pustules along the side of the nose, which were recognised as being connected with glanders. Mr. Smith procured some of the matter, and inoculated an ass, which in the course of four or five days became sick, and was subsequently attacked with glanders. The same phenomena as observed in the latter case of inoculation were present; the cartilages of the joints were also found to be ulcerated. Dr. Hutton exhibited some drawings to show the condition of the parts. He also exhibited a drawing of a case which had occurred some years back at the Richmond Hospital under the care of the late Dr. M'Dowell, before the disease was sufficiently known. The drawing had been made by Mr. Conelly, and Dr. Hutton observed that he had represented the features of the disease with great accuracy, and had depicted most faithfully the white areola which encircles the vesicles. Since that period the areola has been invariably found to be present in every case, and is regarded as one of the pathognomic features of the disease. It has been noticed independently by Dr. Hutton, Mr. Adams, and other observers, and forms one of the marks by which the disease is distinguished from phlebitis. Dr. Hutton said that he should next proceed to read the notes of a case which had recently come under his observation. The patient, J. Butler, a boy about five years of age, was admitted into the Richmond Hospital on the 13th of Dec., 1840. It was stated that he had been always a fine healthy child up to the period of his illness. He complained at first of sickness and pain in his bowels, and on the following day had pains in his knees. About three days afterwards the left side of the face and eyelid became swollen, and the usual symptoms of irritative fever set in, accompanied with thirst, restlessness, quick pulse, and scanty urine. On the 5th of December the fever was increased, and the other side of the face was involved in the swelling; on the 7th a number of pimples with white tops appeared on the inflamed surface. On the 13th, the date of his admission, his face was greatly swelled and inflamed, and presented a number of pustules mixed with several ash-coloured ulcers: he had also an eruption of pustules over his body. Some of these were flattened and somewhat vesicular, like chicken-pock, some were conical and pustular, some in a state of incrustation. Around some of them, particularly those which were in the earlier stage, the peculiar white areola was still visible. Several of the joints were swelled and painful, and there was evident effusion into the left elbow-joint. The child was extremely feverish and irritable, tossing about the bed, and raving: the smell from his body was extremely offensive. He continued in this way, with little change in his symptoms, until the 16th, when he expired. All that could be learned of his history was, that the father was a labourer, and kept a horse, which was said to be labouring under a discharge from his nostrils, the result of cold, but Dr. Hutton said he had not as yet examined the animal. On examination after death, there was an effusion of pus discovered in the left knee-joint. In the thorax there was a small collection of pus close to the edge of the left lung. The lung was of deep red colour, and presented several ecchymosed spots on its surface, and contained two small abscesses. The right lung presented a few flattened tubercles. These were pointed out to the attention of the meeting by Dr. Hutton.—*Dublin Journal of Medical Sciences.*.

---

*Two cases of dislocation of the tendon of the long head of the Biceps Humeri, from its groove.* By John Soden, Jun., Esq.—The first case is that of a man of advanced years, who injured his right shoulder by falling upon his elbow; in six months afterwards he sustained a second accident, a compound fracture of the skull, of which he died; and an opportunity was thereby afforded for examining the nature of the first injury. The symptoms of the injury of the shoulder were always obscure, on account of an alteration in the relative positions of the bones of the joint, which did not apparently depend on a fracture, and could not be considered to amount to a partial dislocation, to which, however, it appeared to be more closely allied than to any other known injury.

The joint was flattened at the posterior and outer parts, and the head of the humerus was unduly prominent in front, and closely drawn up in contact with the under surface of the acromion, grating against it on motion, and becoming locked with it by the upper edge of the greater tubercle striking against that of the acromion on abduction of the arm.

The underhand motions were not much interfered with, except that the patient had no power to raise any object from the ground, on account of the severe pain induced by exercise of the biceps muscle. On examining the joint the accident was found to be a dislocation of the tendon of the biceps from its groove, unaccompanied by any other injury. The joint exhibited extensive traces of general inflammation, and the capsule was thickened and contracted.

The author infers that the altered position of the bones was dependent on the displacement of the tendon, and he explains its influence in the following manner:

The head of the humerus being placed on an almost flat surface, and not inclosed in a bony cavity, is subject to the control of the capsular muscles, which invest it on three sides. These muscles may be said to arise from the upper three-fourths of the circumference of a circle, to the centre of which, represented by the head of the humerus, they converge.

To enable the bone to maintain its equilibrium, it is necessary that the capsular muscles should exactly counterbalance each other; and as there is no muscle from the ribs to the humerus to antagonize the upper capsular muscles, it is suggested that this office is performed by the singular course of the long tendon of the biceps, which, by passing over the head of the bone, when the muscle is put in action, tends to throw the head downwards and backwards; it follows, therefore, that the tendon being removed, the head of the bone would rise upwards and forwards.

Allusion is then made to the frequency with which injury of the tendon is involved, in accidents to the shoulder-joint. A paper by Mr. Gregory Smith, in the 14th vol. of the Medical Gazette, on the "Pathological Appearances in seven cases of injury of the shoulder," is quoted to show, that in all those instances which were accidentally met with in the dissecting room, and are consequently without histories attached, the tendon was either ruptured or displaced; and the same altered position of the bones, as in the present case, was noticed in some of them.

The subject of partial dislocation of the humerus is next considered with reference to the probability of an injury of this tendon being involved in the production of that accident. Only three dissections of partial dislocations are on record; they are to be found in a paper, by Mr. Hargrave, in the Edinburgh Medical and Surgical Journal. One fell under the observation of Mr. Hargrave himself, and the others he quotes from Sir Astley Cooper's large work, and from Dupuytren's Lecons Orales. In Mr. Hargrave's case, the tendon was ruptured; in Sir Astley Cooper's it had been, but had subsequently become reunited; and in Dupuytren's its condition is not mentioned.

The second case is one of a man, who, among other injuries, sustained a dislocation forwards of the humerus. Great difficulty was experienced in the reduction; and after death, for the man only lived for a few days, the joint was examined: it was found that the tendon was dislocated, and that it had passed completely over the head of the bone on its inner side, and was lying at the back of the joint. The author attributes the difficulty of reduction to this complication, with the displacement of the bone.

*On absorption and regeneration of the neck of the thigh-bone after fracture within the capsular ligament.* By W. W. Beever, Esq., of Manchester.—The patient, a woman aged seventy-three, lived nearly four years after the accident. On examination, no vestige of the neck remained, except a triangular portion of the under surface, three-fourths of an inch in length, which, from the obliquity of the fracture, had not been detached from the head. This was articulated by a distinct capsule to a second fragment jutting out from the shaft, and firmly united to it immediately anterior to the lesser trochanter. This adventitious joint, and a band of ligamentous structure extending from the posterior edge of the head to the capsular ligament, formed the only connection between the head and body of the femur. From the large quantity of callus thrown out by the trochanter and head of the bone, the author infers the possibility of bony union being effected.

*An account of two cases of Aneurism of the Trunk of the Superior Mesenteric Artery, in one of which Jaundice was induced by Pressure of the Sac.* By James Arthur Wilson, M, D. Physician to St. George's Hospital.—The symptoms, which had most attracted attention during life in the first of these cases, had been very severe pain between the shoulders along the track of the sixth or eighth lower dorsal vertebræ. The patient died, after illness of about six months, in a state of great exhaustion, much aggravated by mercurial salivation.

On examination of the body a large globular tumor was seen extending from behind the head of the pancreas upwards, forwards, and outwards to the right side. The ductus communis was in close contact with this sac, but was, however, pervious to a probe. The poribiliarii of the liver were universally much enlarged.

The heart was small: the membrane lining its cavities uniformly yellow. Tubercle of a consistence like mortar, and of a yellow colour, were observed in the lungs. In the head the dura mater was universally yellow; but both tunica arachnoidea and pia mater were free from that colour. The substance of the brain was also normal in colour; but a thin yellow fluid could be pressed from the divided surfaces of many of the vessels. The synovial fluid contained in cavities of joints was yellow: their cartilages were of the normal colour. The stomach contained thick yellow mucus.

The author observes that this case may lead us, under similar circumstances, to apply the ear to the upper part of the abdomen as a means of inquiry; it may also prevent our being taken by surprise in the event of sudden death: he also remarks on the inefficiency of the mercurial treatment adopted. In the other case noticed by Dr. Wilson there was a tumor pulsating in the epigastric region, about the size of a small orange, which, when the patient lay flat, projected to the left of the scrobiculus cordis. When the patient turned to the left side, the tumor ceased to be perceptible. On his turning to the right it might again be observed. Between Feb. the 11th, when he was admitted, and July the 12th, when he died, he was attacked with frequent hæmoptysis; and towards the last symptoms of phthisis presented themselves. In the course of this illness there was severe and increasing pain down the dorsal vertebræ, and cramps in the legs; and the tumor became more and more tender to the touch.

The aneurism in this case was in the trunk of the superior mesenteric; it was large, and kidney-shaped, raising up with it the pancreas, which lay at the upper extremity of the tumor.

The author notices, as distinguishing points in these two cases of aneurism of the superior mesenteric artery, that jaundice was, during life, a symptom of the one,—hæmoptysis of the other. In the latter case, the lungs, he observes, were extensively diseased by tubercles of the common kind.—*Trans. Royal Med. and Chirur. Society, in London Med. Gaz.*

---

*Rupture of the Bladder from Retention of Urine.*—J. D., aged 70, was admitted on May 17th, under the care of Mr. Liston. He came into the hospital at ten A. M., on account of a slight transverse wound of the throat, which he had made with the intention of destroying himself. He assigned as a reason for this proceeding, that he had not passed any water since the 14th, although several attempts had been made to pass the catheter by a surgeon. On examination, Mr. Taylor, the house-surgeon, found the bladder greatly distended, and reaching nearly to the navel, with extreme pain on pressure; but the pain was restricted, or nearly so, to the hypogastrium. On farther inquiry it appeared that he had had stricture for several years, but had never before experienced a complete stoppage. Mr. Taylor immediately passed a No. 5 catheter, and met with a stricture three or four inches from the orifice; from eight to nine ounces of turbid urine followed. The pain and tenderness, however, continued without any relief, and at four P. M., it was first remarked that there was increased fullness of the hypogastrium, with tension and acute pain on pressure extending to the general surface and sides of the abdomen. Eighteen leeches were ordered to the hypogastrium, to be followed by hot fomentations; and as the bowels had been confined, ten grains of calomel, followed by half an ounce of castor oil, were administered.

7 P. M. The swelling and tension not at all relieved; tenderness somewhat increased; pulse full, 110; great thirst. He was bled to twenty ounces, and a fresh supply of leeches were ordered to the abdomen: five grains of calomel and five grains of Dover's powder every four hours.

May 18. Passed a restless night; has vomited some bilious matter this morning; pain and tenderness much aggravated, and patient's appearance greatly altered; is affected with clammy sweats; his respiration is hurried, and there is loud sonorous rattle over the whole chest. Bowels were freely opened during the night.

9, P. M. As little urine had been observed to pass since the introduction of the catheter, that instrument was again introduced. Not a drop of urine followed.

19. Patient is rapidly sinking, is quite unconscious. Died at five, P. M.

*After-death Appearances.*

The examination was made twenty-four hours after death.

*Abdomen.*—The parietal portion of peritoneum anteriorly in a state of intense inflammation, being nearly of a black hue; this appearance extended above the umbilicus. Considerable effusion of dark and turgid serum into the cavity, having no ammoniacal smell. Intestines, omentum, and mesentery, also much injured. On displaying the bladder, which was contracted, its serous covering was found intensely inflamed and adherent posteriorly to the rectum. On removing the serous tunic, the cellular tissue around and beneath was found to be infiltrated with a bloody serum, having a strongly urinous odour; the cellular membrane broke down readily under the fingers.

The infiltration has extended throughout the pelvis, reaching nearly to the kidneys. The whole of the urinary organs were now removed, together with the symphysis pubis.

As rupture of the bladder was evident, air was forced into it by the ureter, and found to escape at the back part where the serous coat was bulged out into a pouch. On laying it open, about two ounces of thick urine, and a small calculus, about the size of a peppercorn escaped. The mucous membrane was pale, but much sacculated, forming several small cysts between the fasciculated muscular coat. Posteriorly there was a round sloughy patch of the size of a shilling, communicating by a small aperture with the external cellular membrane, which was also in a sloughy state. The neck of the bladder presented a fringe of warty caruncles over the uvula. There was slight enlargement of the middle lobe of the prostate. On laying open the urethra, at the situation of the stricture, there was found to be a warty thickening of the mucous membrane about an inch in extent. The kidneys were pale and flabby, with a slight appearance of granular degeneration.

*Thorax.*—The lungs were congested posteriorly, with signs of intense bronchitis.

*Heart* enlarged and flaccid, with ossific deposit at the base of the mitral and aortic valves.

*London Lancet.*

---

*Specimen of the effect of a Ligature upon the Femoral Artery.*—Professor Harrison said he had lately had an opportunity of examining the artery of a man who had been operated on for popliteal aneurism about eight or nine years ago in the Richmond Hospital. The case had been under the care of the late Dr. M'Dowell, who took up the artery in the upper third of the thigh, and the man was discharged, cured, in the course of a few weeks. He continued to enjoy good health until a short time since, when he was attacked with bronchitis, for which he was taken into Sir Patrick Dun's Hospital. At the time of his admission he was in a state of great exhaustion, and survived only a few days. On dissection, the lungs were found to be emphysematous, and exhibited the anatomical characters of bronchitis. There was nothing remarkable in the heart, except some hypertrophy of the right ventricle, but the aorta showed numerous traces of organic alteration. It retained its cylindrical form, without any tendency to collapse of its walls, and at some points the sides of the vessel could not be approximated without considerable force. Its interior was found to be thickly studded with calcareous scales, covered in most places by the lining membrane, but in some the lining membrane was deficient over the calcareous deposit. At the place where the ligature had been applied upon the femoral artery, there was obliteration of the canal of the vessel to the extent of an inch and a half. Half an inch above this obliterated portion, the profunda artery arose, and appeared to be considerably dilated. Below it the femoral artery was pervious down to the ham, where it again became obliterated to the extent of about two inches. The articular arteries were enlarged.—*Dublin Journal of Medical Science*, July, 1841.

---

*Two Cases of Goitre cured by Ligature.* By M. J. Bach, of Strasbourg.—Henri Klein, aged 30, first perceived an enlargement in the front of the neck, about ten years ago, which gradually but slowly enlarged, until it attained such dimensions as to become very troublesome from its pressure on the larynx and trachea. The tumour was moveable, though adherent by a pretty broad base. It felt at certain points hard, almost cartilaginous or osseous, whilst in others it was soft and presented fluctuation. It extended upwards to within two finger-breadths of the ramus of the lower jaw, and downwards to about the same distance from the clavicle, displacing outwards the sterno-mastoid muscle and carotid artery on the right, and pushing the larynx inwards and towards the left.

M. Bach having resolved to free him of this tumour by operation on the 25th of July, made a crucial incision over the centre of the tumour, and, after dissecting back the flaps of skin thus formed with his fingers, broke down all the cellular attachments of the tumour, and turned it out, leaving it still, however, attached by its vascular base. Around this a strong ligature was placed and moderately tightened, the ends of the ligature being passed through a silver tube, in order to admit of the pressure being increased as required. Lint dipped in cold water was then laid over the tumour, and was directed to be frequently renewed. The patient was then put to bed.

The constriction was twice increased during the day as the ligature became looser, but was never drawn tight. At each time when the ligature was tightened, pain in breathing and swallowing was complained of, as well as a pain which extended towards the nape of the neck.

On the 26th, pretty intense reaction had come on, requiring blood-letting, and the tumour had become of a black colour. Puncturing it with a needle gave no pain. The ligature was twice tightened on this and the following days.

On the 31st, the tumour had become moveable, and emitted a strong gangrenous odour. It was therefore cut off before the ligature, leaving this still in its place, and tightening it still further.

By the 5th of August the pedicle had completely disappeared in consequence of the free suppuration which had been established, the flaps of skin were therefore brought in contact over the wound by means of adhesive plaster, and by the end of the month he was discharged cured.

In the other case the man was 27 years of age, and the tumour about the size of an orange. Instead of the crucial incision, a simple transverse incision was made, the tumour turned out in the same way, and the ligature passed around its root, gradually and daily tightened. On the fourth day the tumour was cut off before the ligature; on the sixth day the ligature itself was removed; and by the thirtieth day after the operation he was discharged cured.

M. Bach considers that all goitres are not curable by operation. When the enlargement of the thyroid body depends on the presence of cysts in its substance, or when it is simply enlarged, as in simple goitre, or even when it presents a scirrhous induration, provided in all these cases the adhesions are not too extensive, or the base too broad, M. Bach thinks they may with propriety be operated on. In that form which he denominates the aneurismal, and in that depending on development of the parenchymatous structure, he recommends that no operation be attempted, the first being beyond the reach of art, the other yielding to medical treatment. He recommends the ligature in preference to the knife in every case, and urges the necessity of making the pressure very moderate, for the first three days at least. In this way, he says, acute pain is avoided, and the patient is not exposed to violent fits of suffocation, nor to hæmorrhage from the ligature cutting through the morbid vessels, nor to phlebitis, symptoms which are but too apt to come on if this be not attended to.—*Ed. Med. and Surg. Journal, from Gaz. Med. de Paris*, 2d January, 1841.

---

*On the utility of Oxalic Acid in Inflammations of the Mucous Membranes.* By M. Nardo.—At the scientific meeting at Turin in September last, M. Nardo made known the results of his experiments on the therapeutic effects of oxalic acid; to which subject he had been devoting his attention for the last twelve years. From his experiments he concluded that this acid possesses antiphlogistic properties superior to that of any other vegetable acid, as the malic, the citric, the acetic, or the tartaric, and that, in addition, it possesses the precious property of calming the violent pain which attends inflammation of the mucous membranes. He especially recommends its employment in all diseases where this membrane is implicated, as in angina, gastritis, gastro-enteritis, stomatitis, and aphtha. He says that the use of oxalic acid renders the loss of blood much less necessary. The dose he emyloyed was one and a half grains in about eight ounces of fluid. It is not mentioned how often it ought to be repeated. He regards it as a contra-stimulant. —*Ibid., from Repertorio delle Scienze Fisico-Mediche del Piemonte.* January, 1841.

---

*Further facts regarding the identity of Small-Pox and Cow-Pox.* By Dr. Basile Thiele, of Kasan in Russia.—In the spring of 1836, Dr. Thiele inoculated the matter of small-pox on the udder of some cows; pustules were produced which bore all the characters of the true vaccine vessicle in those animals. The matter from these vesicles were applied by punctures to the arms of several children, and produced the true vaccine disease; only that the local symptoms were more intense, and the consecutive fever more severe. The vaccine lymph thus procured was carefully observed during seventy-five successive transmissions in the human subject, and appeared always to retain its normal character.

During the spring of 1838, M. Thiele repeated his former experiments with the same success. The cows chosen were from 4 to 6 years old, and they were inoculated with the small-pox matter on the posterior part of the udder. Whether the matter used was taken from mild or severe, from the single or the confluent form of small-pox, the vesicle produced on the udder of the cow was always of the same mild character, and the lymph obtained from it proved equally mild when applied to the human system. In general only a third of the inoculations succeeded. On the third day, slight induration of the cellular tissue at the seat of the puncture was observed. On the fifth a pustule formed; from the seventh to the ninth it had the appearance of a vaccine vesicle, from the ninth to the eleventh it began to desicate and form a crust, which fell off a few days after, leaving a deep cicatrix; from the fourth to the seventh day the circulation of the animal was slightly accelerated, and its temperature increased, but its general health appeared to remain unaltered.

Dr. Thiele satisfied himself, from experiment, that the virus thus obtained from the cow ought to be allowed to remain dry on glass for five or six days before being used, and then mixed with a little fresh warm milk before being applied to the arm of the infant. The vesicles resulting from the vaccination of this lymph appeared in every respect similar to those of the ordinary vaccine vesicles. During the first transmissions through the human subject, Dr. Thiele observed two separate febrile attacks; one from the third to the fourth day, and the other from the eleventh to the fourteenth day; in this respect still bearing some analogy to small-pox. He says that this double febrile accession is not got the better of till about the sixth transmission, and that it is not till then that it is safe to give up mixing the lymph with warm milk previous to vaccination. To the neglect of this precaution he attributes the occurrence of some cases of true small-pox eruption after vaccination, with this lymph during its earlier transmissions; but he immediately afterwards mentions a circumstance which appears to invalidate the statement as to its being true small-pox eruption; for he states some of this matter, taken and mixed with milk, communicated the vaccine disease to other children, and remained as vaccine lymph in all its subsequent transmissions.—*Ibid.*, from *Bulletin de l' Academie Royale de Medicine*, January 1841.

*The Art rather than the Science of Surgery.*—We find from a late number of "L'Experience," that in the short time since the first performance of the operation for squinting, no less than 49 different instruments for its performance have been invented in France and Germany, and are to be found at M. Charrier's. We fear that a considerable addition to their number, if not to their utility, might by made from our own country. The chief inventors are MM. Dieffenbach, Phillips, Jules Guérin, Velpeau, Leroy d'Etiolles, and Lucien Boyer.—*Lond. Med. Gaz.*

